# The Physician’s Visiting List for 1893

**Published:** 1892-12

**Authors:** 


THE PHYSICIAN'S VISITING LIST FOR 1893.—Forty-second year of its publication. Philadelphia: P. Blakiston, Son & Co. Price, $1.00.

THIS visiting list has been in use many years and the form has become almost a standard. Its publication was first begun some forty-two years ago by Lindsay & Blakiston, and has been published annually since that time. It contains leaves for the regular daily record of visits, accommodating twenty-five patients per week, and a memoranda covering the twelve calendar months, followed by those for addresses of patients and others, nurses; addresses, bills and accounts asked for, memoranda of wants, obstetric engagements, vaccination engagements, obstetric cases, record of deaths and cash account. The U. S. Pharmacopœia for 1890, which will shortly be published, adopts in a great measure the metric system of weights and measures; this will doubtless create much confusion in the minds of physicians and druggists and lead to many misunderstandings and errors. In order to provide a guide to the proper dosage, etc., Dr. Geo. M. Gould, author of the New Medical Dictionary, has prepared a very complete table of the officinal and unofficinal drugs, with doses in both the metric and English systems; this table is published in this little book, together with a short description of the metric system, also some notes upon the metric or French decimal system of weights and measures, by Oscar Oldberg, Pharm. D., table for converting apothecary's weights and measures into grams, posological table, a list of new remedies, incompatibility, poisons and antidotes, disinfectants, examination of urine, forms of Bright's disease, diagnosis and treatment of the simpler superficial diseases of the eye, the eruptive fevers, asphyxia and apnœa, comparison of thermometers, table for calculating the period of utero-gestation and local therapeutics. C. E. J.

